# Engineering Peptide-Based Polyelectrolyte Complexes with Increased Hydrophobicity

**DOI:** 10.3390/molecules24050868

**Published:** 2019-03-01

**Authors:** Sara Tabandeh, Lorraine Leon

**Affiliations:** 1 Department of Materials Science and Engineering, University of Central Florida, Orlando, FL 32816, USA; sara.tabandeh@Knights.ucf.edu; 2 NanoScience Technology Center, University of Central Florida, Orlando, FL 32826, USA

**Keywords:** polyelectrolyte complexes, coacervates, hydrophobicity, encapsulation, polypeptides, self-assembly, chirality

## Abstract

Polyelectrolyte complexation is a versatile platform for the design of self-assembled materials. Here we use rational design to create ionic hydrophobically-patterned peptides that allow us to precisely explore the role of hydrophobicity on electrostatic self-assembly. Polycations and polyanions were designed and synthesized with an alternating sequence of d- and l-chiral patterns of lysine or glutamic acid with either glycine, alanine or leucine due to their increasing hydrophobicity index, respectively. Two motifs were considered for the oppositely charged patterned peptides; one with equal residues of charged and uncharged amino acids and the other with increased charge density. Mass spectroscopy, circular dichroism, H- and F-NMR spectroscopy were used to characterize the polypeptides. Polyelectrolyte complexes (PECs) formed using the sequences were characterized using turbidity measurements, optical microscopy and infrared spectroscopy. Our results show that the critical salt concentration, a key measure of PEC stability, increased with both increasing charge density as well as hydrophobicity. Furthermore, by increasing the hydrophobicity, the amount of PEC formed increased with temperature, contrary to purely ionic PECs. Lastly, we assessed the encapsulation behavior of these materials using a hydrophobic dye. Concluding that encapsulation efficiency increased with hydrophobic content of the complexes providing insight for future work on the application of these materials for drug delivery.

## 1. Introduction

Interactions between oppositely charged macromolecules in solution lead to polyelectrolyte complexes (PECs) that phase separate into materials that behave either as a liquid or a solid [1,2,3,4,5]. The liquid-liquid phase separation is termed complex coacervation which initially forms polymer-rich droplets that eventually coalesce into a distinct phase. [2,3] The term was introduced by Bungenberg de Jong et al. [6], based on their work on ionically interacting polymers in aqueous media. Complex coacervates have extremely low interfacial tension with water [7,8] which has led to encapsulation applications in the food industry [9,10] as well as potential applications in the pharmaceutical industry [11,12,13,14]. Moreover, coacervates are being explored as potential vehicles for gene delivery [15,16,17], as adhesives [18,19], as bio/nanoreactors [20,21] and as a mechanism for the formation of membraneless organelles [22]. The dominant driving force for coacervation has been determined to be an increase in entropy from counterion release that occurs when two oppositely charged macroions come together [23]. 

PECs that form solids (precipitates), have glassy behavior, where the glass transition temperature has been shown to be dependent on the number of water molecules surrounding a polyelectrolyte-polyelectrolyte intrinsic ion pair [24]. In general, strong electrostatic attractions lead to solid precipitate formation, while weak pairs of polyelectrolytes are more likely to form complex coacervates [25]. Apart from the electrostatic interactions, shorter range forces like hydrogen bonding have been also found to control the physical state of the polyelectrolyte complexes [2,4]. These solid complexes have not been as extensively explored in comparison to their liquid counterparts. This is likely due to the non-equilibrium nature of the strong interactions leading to kinetic effects that make reproducibility difficult as well as the brittle nature of the materials. However, using salt as a plasticizer has allowed processing via compaction, extrusion, and ultracentrifugation [26]. 

Charged polypeptides provide unique opportunities for PEC-based materials due to their biocompatibility, ability to form secondary structure, and sequence specificity [23]. This sequence specificity can provide a robust platform to study PEC behavior. For instance, Chang et al. designed a series of lysine-glycine (KG) copolymers in which the distance between charged amino acids was varied, but the overall charge of the molecule remained the same. Both experimental and theoretical results, determined that phase separation was increased with increasing charge separation or blockiness due to a greater entropic contribution from counterion release when the ions were initially more confined in a block [27]. Chirality of the amino acid monomers has been demonstrated as an important factor that affects physical properties of polypeptide-based complexes [28]. In a study combining experiments with molecular simulations [2], homochiral polypeptides were found to form hydrogen-bonded precipitates resulting in β-sheet formation while racemic polypeptides formed liquid coacervates as their chirality patterns disrupt backbone hydrogen bonding. During β-sheet formation, chains bond together closely, and the resultant packed structure has less mobility and water content. On the other hand, racemic peptides with their randomly alternating chirality hinder hydrogen bond formation, forming liquid coacervates with higher amount of water and more mobility [28]. Follow up work using chiral patterned peptides explored the effect of sequence, determining that 8 or more consecutive L-amino acids in a peptide chain resulted in solid complexes with β-sheet FTIR signals when mixed with an oppositely charged peptide containing the same pattern [3]. Interestingly, intramolecular hydrogen bonding, in the form of α-helix formation, has been shown to increase PEC stability compared to non-helical peptides of the same molecular weight, but has no influence on phase behavior. The PECs formed complex coacervates regardless of the chirality of the oppositely charged polypeptides since the hydrogen bonds were already occupied in helix formation. The enhanced stability was due to an increase in charge density as the polypeptide folded. This was demonstrated using ultra-stable helical peptides designed to maintain their charge and retain their secondary structure, unlike polylysine and polyglutamic acid [29]. 

Beyond chirality, polyelectrolyte complexation is affected by stoichiometry of mixing (ratio of polycation to polyanion), total polymer concentration, pH, charge density and molecular weight of the polymers, the ionic strength of the solution, and temperature [28,30,31,32,33,34]. For instance, pH of the solution can change the ionization degree of the charged groups and therefore, the amount of electrostatically-driven complex formation [33]. Generally, the maximum amount of complex formation is found at charge neutrality [32,35]. High ionic strength decreases complexation via the interaction of salt microions with the charged groups on the polymer chains, decreasing the effective polymer interactions with each other [35,36]. The electrostatic and non-coulombic interactions can be influenced by temperature through interactional parameters and conformational changes [33]. Increasing the charge density produces more available sites per chain length and, more complexation would be expected [29]. Likewise, higher molecular weight and total polymer concentration have been shown to increase PEC formation [32]. 

Hydrophobicity, another parameter that can play an essential role in complexation, has been less understood. It can affect polyelectrolyte associations together with the electrostatically-driven interactions, both, by their impact on water structuring around the site of interaction [37]. When hydrophobic molecules are placed in water, the water molecules form a cage-like structure at the interface of the hydrophobic molecule. Thus, the driving force for hydrophobic associations is an increase in water entropy. Similarly, as a consequence of water destructuring, entropy would increase and provide a greater driving force for complexation. Sadman et al. [38], explained hydrophobicity as a powerful parameter to tune the properties of PECs. They compared swelling and mechanical behavior of the hydrophobic complexes with a hydrophilic complex of poly(styrenesulfonate)/poly (diallyldimethylammonium) using the quartz crystal microbalance (QCM). They increased the hydrophobicity of quarternized poly(4-vinylpyridine) (as the cationic side) by increasing its side chain length using methyl, ethyl and propyl substituents, each to be paired with poly(styrenesulfonate). They showed that there was a difference in swelling behavior with degree of hydrophobicity, where more hydrophobic complexes absorbed less salt solution. Interestingly, the mechanical properties were found to depend on overall water content within the PEC, which could be tuned via hydrophobicity and the nature of the salt. This swelling behavior has also been observed in a study by Mende et al. [39], on strong polyelectrolytes based on alternating maleic anhydride copolymers, where the formation of less swollen particles with the more hydrophobic polyelectrolytes using atomic force microscopy was reported. 

In this paper, we aim to examine the role of hydrophobicity on polyelectrolyte complexation using peptide-based polyelectrolyte complexes. Solid phase synthesis allows for precise positioning of monomers in specific patterns. Therefore, unlike previous studies, we can introduce entirely hydrophobic monomers, in conjunction with our charged monomers in the design of our polyelectrolytes. This will allow for a careful characterization of the effects of both hydrophobic patterning and overall hydrophobicity on the properties of PECs. In addition, we explore the ability of these materials to encapsulate small hydrophobic molecules, with the aim of future use of these complexes as delivery carriers for both charged therapeutics such as nucleic acids or proteins and hydrophobic drugs. 

## 2. Results and Discussion

### 2.1. Peptide Design

We have used rational design to engineer molecular sequences of polypeptides which have different hydrophobic content in order to explore the effect of hydrophobicity on complex formation. As mentioned earlier, the use of racemic sequences of polypeptides can lead to coacervate complex formation due to the prevention of hydrogen bonding by the peptide structure [2]. Therefore, an alternating sequence of L and d-chiral amino acids was chosen for the patterned peptides to promote liquid coacervate formation due to their easier processability and various proven applications. Two patterned sequences were designed; the first pattern includes (kX) as the cationic molecule and (eX) as the anionic molecule, the second pattern with the purpose of increasing charge density and evaluating of its effect on complexation includes (kKx) as the cationic molecule and (eEx) as the anionic molecule of the complex pairs. K refers to lysine and E to glutamic acid. Lower and upper cases show d- and l-chirality, respectively. Glycine (G), alanine (A) and leucine (L) were chosen for the X position due to their increasing hydrophobicity index, respectively [40]. Enhanced hydrophobicity can be expected by the longer aliphatic side chain from glycine to alanine and leucine (glycine < alanine< leucine, see Table 1). Patterned peptides are summarized in Table 2. The molecules were synthesized using solid phase peptide synthesis. 

### 2.2. Characterization of Polypeptides

In order to confirm the molecular structure and chiral pattern of the synthesized polypeptides ^1^H nuclear magnetic resonance (NMR) and F-NMR spectroscopy, MALDI-TOF mass spectroscopy and Circular Dichroism (CD) were used. 

#### 2.2.1. Matrix-Assisted Laser Desorption Ionization-Time of Flight (MALDI-TOF) Mass Spectroscopy 

The measured mass to charge ratio (*m*/*z*) in MALDI-TOF spectroscopy can verify the accuracy of the peptide synthesis. Mass spectroscopy results are summarized in Table 3 and the collected spectra are shown in Supplementary Figure S1. The actual molecular weight was approximately equal to the calculated mass for all the peptide sequences, confirming the desired polymer synthesis process and that all peptides are composed of 30 amino acids. The small variation in actual and calculated molecular weight could be due to the presence of counterions. 

#### 2.2.2. Proton Nuclear Magnetic Resonance (H-NMR) Spectroscopy

The chemical structure and ratio of amino acids in a structural unit of peptides can be verified by H-NMR spectroscopy. The area under a signal in the H-NMR spectrum can determine the relative number of hydrogens which that signal represents. The location of the α-carbon’s hydrogen appears around 4 ppm. The structural unit of the first generation of patterned peptides consists of two amino acid residues, (kX) and (eX), whereas the second generation formed by three amino acid residues, (kKx) and (eEx). Analysis of the H-NMR spectra (Supplementary Figure S2) confirms the ratio of 1:1 for k:X (or e:X) of the first generation and 2:1 for kK:X and eE:X for the second generation. All other hydrogens in a structural unit of the peptide sequences are also shown on the spectra in Supplementary Figure S2. A residual solvent signal attributed to diethyl ether (at around 1.17 and 3.56 ppm for the CH_3_ and CH_2_ protons of diethyl ether, respectively), which was used in the peptide precipitation process after the cleavage, can be observed in the H-NMR spectra of some samples. This signal can be successfully removed using dialysis or multiple lyophilization processes. We have confirmed this by repeating the H-NMR measurements of some samples after dialysis or three times lyophilization. Supplementary Figure S3 shows two typical comparisons of the solvent peaks before and after three times lyophilization for p(kG) and before and after performing dialysis for p(eEg).

#### 2.2.3. Circular Dichroism (CD) Spectroscopy

CD measures the difference in the absorption of left and right-handed circularly polarized light. For this reason CD can be used to confirm different chiral patterns of polypeptides, in addition to measurement of secondary structure motifs. For our first generations of polypeptides, kX and eX, an alternating sequence of D and L amino acids have been used (except for glycine which does not have chirality). As shown in Supplementary Figure S4a,b, both p(kG) and p(eG), have a maximum near 195 nm. Since glycine is achiral and both lysine and glutamic acid have D-chirality this maximum at 195 nm is indicative of a random coil configuration, normally manifested as a minimum for peptides with L-chirality. For kA, kL, eA, and eL (Supplementary Figure S4a,b the CD spectra is lower in magnitude, since contributions of L and D amino acids are opposite and additive. p(kKx) and p(eEx) peptides (Supplementary Figure S4c,d) show a nearly flat absorbance signal due to the adjacency of two identical amino acids of opposite chirality (kK and eE) that cancel out. This flat CD spectra has been observed before for polypeptides of alternating L and D chirality [3]. 

#### 2.2.4. Fourier Transform Infrared (FTIR) Spectroscopy

**Figure 1:**
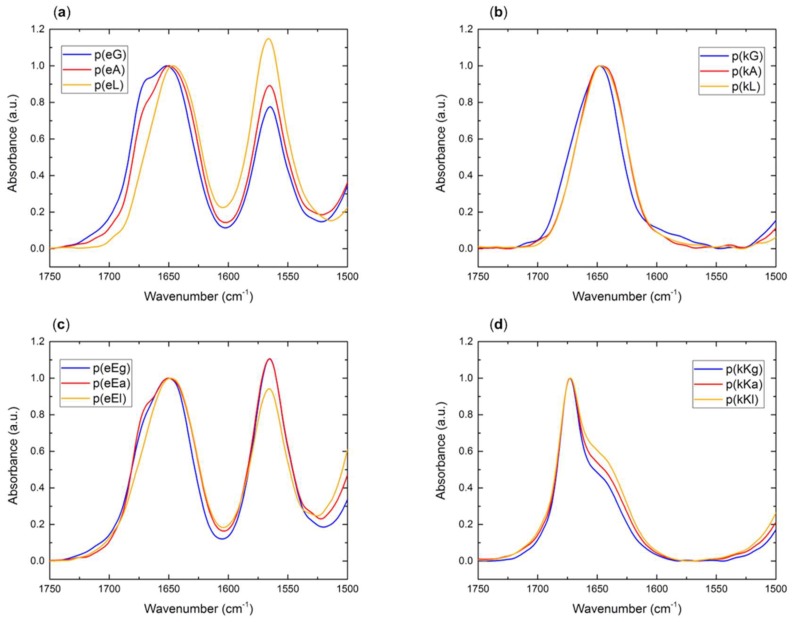
FTIR spectra of the amide I region of the hydrophobically patterned peptides at a concentration of 100 mM of charged monomer: (**a**) Polyanions of (eX) sequence; (**b**) Polycations of (kX) sequence; (**c**) Polyanions of (eEx) sequence; (**d**) Polycations of (kKx) sequence, containing a TFA counterion peak at 1673 cm^−1^. Data is normalized. All spectra have a peak at 1645 cm^−1^ indicative of a random coil. All polyanions contain a peak at 1564 cm^−1^ attributed to the side chain carbonyl stretch of glutamic acid.

Secondary structure analysis of individual polypeptides and PECs can be analyzed using FTIR spectroscopy. Characterization of polypeptides is determined by the location of the amide I carbonyl stretching vibration in FTIR (1600–1700 cm^−1^). Conformation of the hydrogen bond in which the carbonyl groups are involved affects the absorption wavelength present in different types of secondary structure (i.e., random coil, β-sheet, β-turn and α-helix) [41]. Figure 1a–d, shows the spectra for the individual polycations and polyanions. A peak at 1645 cm^−1^ which is the characteristic of random coil structure [42], is observed for all individual polypeptides. During cleavage in trifluoroacetic acid (TFA), positively charged peptides acquire a TFA counterion which shows a stretching peak at 1673 cm^−1^ [43]. To prevent overlap between the TFA peak and a low intensity β-sheet peak which appears around 1680 cm^−1^ [3,44], we dissolved the kX peptides in a 5 mM HCl solution and lyophilized them and repeated this procedure 3–4 times. To confirm the removal of TFA, F-NMR was taken before and after the procedure as shown in Supplementary Figure S5. As indicated by Figure 1b, there is no peak at 1673 cm^−1^ for the FTIR absorbance of (kX) sequences. The elimination process of TFA was not performed for the second sequence of polycations, (kKx), due to the observation of liquid coacervates in the optical microscopy of their complexes which will be discussed later. A peak at 1564 cm^−1^ attributed to the side chain carbonyl stretch of glutamic acid is observed for all polyanions. 

### 2.3. Stoichiometry and Temperature Effect on Complex Formation 

**Figure 2:**
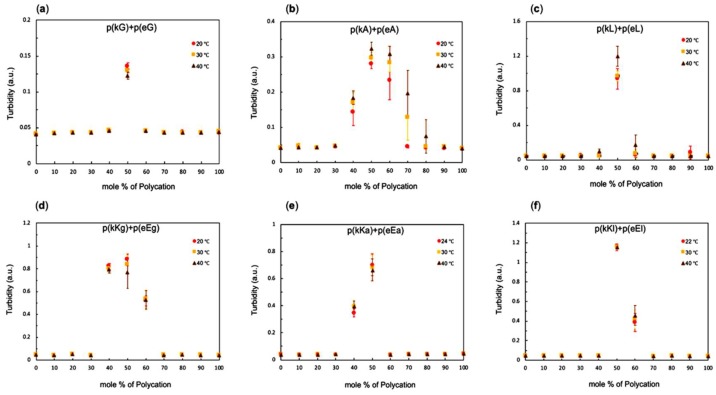
Turbidity of polypeptide mixtures as a function of polycation content (mole%) at room temperature, 30 and 40 °C (5 mM concentration with respect to the total monomer charge; pH of the polypeptide solution was adjusted to 7): (**a**) p(kG)+p(eG); (**b**) p(kA)+p(eA); (**c**) p(kL)+p(eL); (**d**) p(kKg)+p(eEg); (**e**) p(kKa)+p(eEa); (**f**) p(kKl)+p(eEl). All plots show maximum turbidity at 50 mol% polycation. Both (**a**,**d**,**e**) indicate decreases in turbidity with temperature. Both (**b**,**c**) show increases in turbidity with temperature.

Turbidity is a qualitative way to reflect the extent of the complex formation based on the measured light transmission which changes with the size and composition of the complex phase [32,45]. Salt-free complexes were examined by varying the ratio of polycation to polyanion, using a total charged monomer concentration of 5 mM and by varying the temperature. Before mixing polypeptide solutions were adjusted to pH = 7 in order to be fully charged. Turbidity results for p(kX)+p(eX), as well as p(kKx)+p(eEx), as in Figure 2, indicate the highest turbidity at 1:1 ratio of polycation to the polyanion for all complexes. The extent of complexation depends on the amount of oppositely charged groups in solution, since both polypeptides have the same number of charges therefore, maximum complexation is achieved at an equimolar ratio [46,47]. 

Figure 2 also shows turbidity values for the different complexes as a function of temperature. The effect of temperature on polyelectrolyte complexation in the absence of hydrophobic interactions can be rationalized using Flory-Huggins interactional parameters. The χ parameter between solvent and polymer increases as temperature decreases (χ ~ 1/T) favoring demixing or phase separation. The χ parameter between the two polymers decreases as temperature decreases which also favors interactions between the polymers. However, the interactional parameter between two polymers is the leading cause due to the greater size of the polymers compared to the solvent [33]. On the other hand, hydrophobic interactions are enhanced at higher temperatures [33]. Investigation of the effect of increasing temperature on PEC formation of salt-free systems by turbidity indicates a decrease in complex formation of p(kG)+p(eG) (Figure 2a) verifying the fact that electrostatic interactions are the driving force for complex formation while the two other systems of the same sequence with alanine and leucine show higher turbidity values at increased temperatures confirming the increase in hydrophobic interactions. However, temperature dependency can be observed only for the ratios close to equal stoichiometry. Related studies using polypeptides did not show any temperature effect on complexes of polylysine and polyglutamic acid at any stoichiometric ratio and explained that by strong electrostatic interactions between oppositely charged polypeptides which have not been affected by the temperature range of 20–40 °C [32]. However, in other work on poly(acrylic acid) and poly(allylamine hydrochloride) complexes, a pronounced temperature effect was observed with the most substantial decrease of turbidity at equal stoichiometry at higher temperatures [46]. 

Complex turbidity of p(kKg)+p(eEg) and p(kKa)+p(eEa) systems decrease in turbidity as the temperature increases which indicates the dominance of electrostatic interactions in these sequences that have higher charge density than p(kX)+p(eX) systems. Interestingly, the p(kKl)+p(eEl) sequences exhibit no temperature dependence at equal molar stoichiometry and a slight increase in turbidity with temperature at 60 mol%. This shows how the increase in the hydrophobicity of the side chain (from 1 carbon to 4 carbons) can change the responsiveness of these materials.

### 2.4. Effect of Ionic Strength and Charge Density on Complex Formation

**Figure 3:**
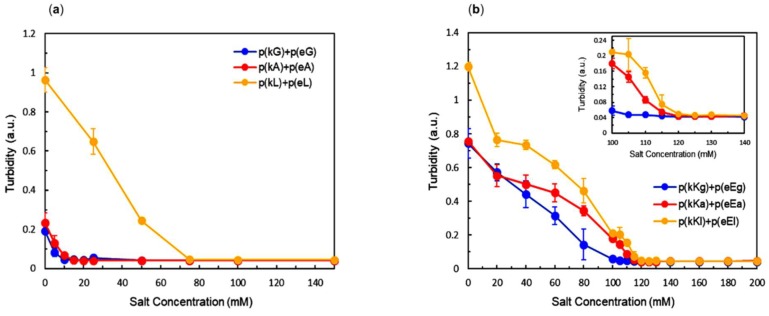
Turbidity measurement as a function of salt concentration: (**a**) p(kX)+p(eX) complexes; (**b**) p(kKx)+p(eEx) complexes. Inset: close up of the range between 100 mM and 140 mM. Error bars are the standard deviation from triplicate measurements.

A stoichiometric ratio of polypeptides at pH = 7 was used to form PECs with a total charged monomer concentration of 5 mM at varied salt (NaCl) concentrations. The change in turbidity as a function of salt concentration is shown in Figure 3a,b. Increasing the ionic strength of the solution can depress complex formation due to the screening of opposite charges of the polyelectrolytes by salt ions [48]. As the salt concentration increases, there will be a point beyond which no phase separation occurs in solution. This point is called critical salt concentration (CSC) [49] and is often used to assess stability of PECs. The p(kX)+p(eX) sequence of peptides shows a low salt resistance with a CSC of 10, 15 and 75 mM NaCl for glycine, alanine, and leucine, respectively (Figure 3a). The sequence with the most hydrophobic character has the highest CSC, illustrating a stabilizing effect on PEC formation. Higher overall turbidity is observed for the most hydrophobic sequence, which tends to decrease with hydrophobicity. This increase in turbidity can be caused by either formation of a larger amount of PEC or a higher amount of PEC in a given volume (higher density and consequently less water). Determining what causes this increase in optical density is a subject of ongoing work. 

**Figure 4:**
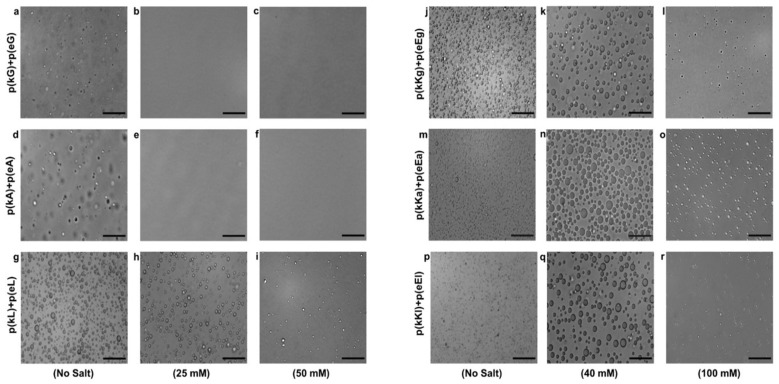
Optical micrographs of complexes at different salt (NaCl) concentrations: (**a**), (**b**) and (**c**) p(kG)+p(eG) at zero, 25 and 50 mM concentration, respectively; (**d**), (**e**) and (**f**) p(kA)+p(eA) at zero, 25 and 50 mM concentration, respectively; (**g**), (**h**) and (**i**) p(kL)+p(eL) at zero, 25 and 50 mM concentration, respectively; (**j**), (**k**) and (**l**) p(kKg)+p(eEg) at zero, 40 and 100 mM concentration, respectively; (**m**), (**n**) and (**o**) p(kKa)+p(eEa) at zero, 40 and 100 mM concentration, respectively; (**p**), (**q**) and (**r**) p(kKl)+p(eEl) at zero, 40 and 100 mM concentration, respectively. Scale bars, 100 µm. Bright field images are taken with a 20× objective.

**Figure 5:**
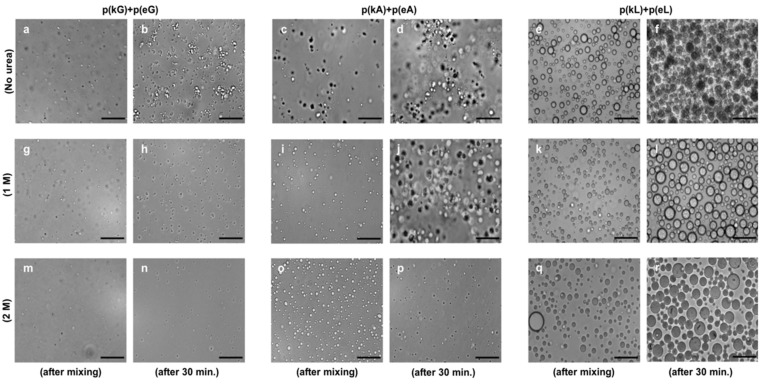
Optical micrographs of p(kX)+p(eX) complexes at different urea concentrations. Scale bars, 100 µm. Bright field images are taken with a 20× objective. Top row (**a**–**f**) at 0 M urea concentration. Middle row (**g**–**l**) at 1 M urea concentration. Bottom row (**m**–**r**) at 2 M urea concentration. Images (**a**,**c**,**e**,**g**,**i**,**k**,**m**,**o**,**q**) were taken right after mixing. While images (**b**,**d**,**f**,**h**,**j**,**l**,**n**,**p**,**r**) were taken 30 min after mixing.

Optical microscopy images are taken to verify the physical state of complexes either being solid precipitates or liquid coacervates (Figure 4). Liquid complexes are characterized as forming spherical droplets with micron sized dimensions, while solid precipitates form irregularly shaped aggregates [2]. Without any added salt, p(kX)+p(eX) PECs show liquid droplet formation at early stages of complexation (just after mixing) as shown in Figure 5a,c,e. Interestingly, these complexes transitioned to more irregularly shaped aggregates after 30 min as shown in Figure 5b,d,f. To evaluate this precipitate-like behavior and the possibility of hydrogen bonding, we studied the stability of p(kX)+p(eX) at both 1 M and 2 M concentrations of urea with the same preparation steps that PECs were examined with salt (Figure 5). Urea can break hydrogen bonds between the chains of polypeptides [2]. It interacts with the peptide backbone (polar amide surface) by engaging in hydrogen bonding and does not affect the hydrophobic association significantly [50,51]. As demonstrated by optical microscopy images (Figure 5), PECs undergo a transition from precipitate-like particles to liquid-coacervate droplets in urea containing solutions. This is clearly shown by the transition of p(kA)+p(eA) complexes from 0 M (Figure 5d) to 2 M (Figure 5p) and the transition of p(kL)+p(eL) complexes from 0 M (Figure 5f) to 1 M (Figure 5k). The transition of p(kG)+p(eG) appears to show the same trend (Figure 5b,h,n) but due to the smaller overall droplet size it is hard to distinguish. This smaller droplet size is attributed to decreased hydrophobic interactions compared to the other sequences. Interestingly, a higher concentration of urea is required for alanine complexes to transition to liquids than leucine (2 M versus 1 M), displaying greater resistance of alanine complexes against hydrogen bond disruption. We believe this is attributed to the overall size of alanine compared to leucine, where the larger leucine side chain disrupts hydrogen bonding in our chiral pattern to a greater extent than alanine. Overall increasing urea concentration tended to decrease the overall amount of PEC formed, this is due to weakening of ion-pair associations in urea [52]. The increase in size of PECs with time can be explained by the tendency of liquid droplets to coalesce in order to reduce their surface contact with water. 

The sequences with higher charge density, p(kKx)+p(eEx), showed a similar behavior of turbidity decrease with increasing concentration of salt but higher overall CSCs were achieved for each complex pair compared to the first generation sequences (Figure 3b). This increase in overall CSC is due to the increased charge density of the sequence which can enhance electrostatically-driven complexation. The CSC for the complex pairs of higher charge density is 105, 115 and 120 mM for glycine, alanine and, leucine, respectively (Figure 3b). The trend of higher CSC with greater hydrophobic content still persists, but is much less prominent for the peptides with higher charge density. Liquid coacervates are the phases formed for p(kKx)+p(eEx) PECs in all conditions having turbidity (Figure 4j–r). The reason for liquid coacervate formation, in these sequences is the presence of the same charged D and L chiral amino acids next to each other in a polymer chain (two lysines in p(kKx) and two glutamic acids in p(eEx)). Work using chiral patterned peptides has already demonstrated that having both of these charged monomers in sequence disrupts hydrogen bonding between oppositely charged molecules [3].

### 2.5. Secondary Structure of Polypeptide Complexes

**Figure 6:**
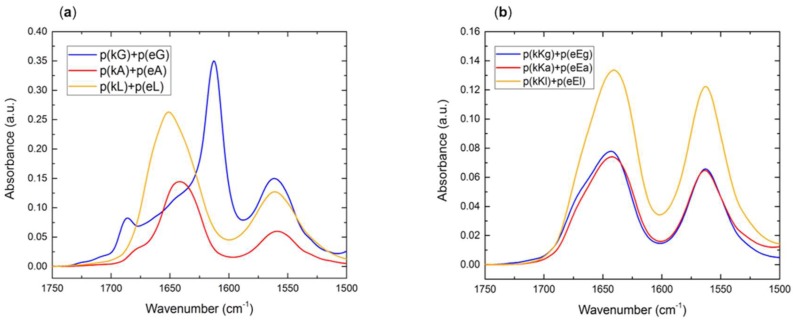
FTIR spectra of polypeptide complexes: (**a**) p(kX)+p(eX); (**b**) p(kKx)+p(eEx).

Structural conformation of polypeptide complexes was evaluated by FTIR spectroscopy, the results of which are illustrated in Figure 6a,b. A superposition of individual polypeptide peaks, including random coil absorbance at around 1645 cm^−1^ and side chain carbonyl stretch of glutamic acid at 1564 cm^−1^ can be observed in each spectrum. The TFA counter ion peak, at 1673 cm^−1^, which was observed for the polycations of the second generation p(kKx), appeared at a very low signal in the spectrum of p(kKx)+p(eEx). The process of TFA elimination from p(kKx) seemed unnecessary due to the formation of liquid coacervates under the studied conditions (confirmed by optical microscopy, see Section 2.3) but the appearance of solid-like particles that could be dissolved with urea for p(kX)+p(eX) complexes, was the reason for TFA elimination, in order to better interpret the FTIR results. Random coil structure is the dominant conformation of the all complexes, as previously mentioned, but appearance of a large peak at 1613 cm^−1^ for p(kG)+p(eG) indicates β-sheet formation [53]. In addition, a low signal peak around 1686 cm^−1^ is observed for p(kG)+p(eG) and a shoulder at 1686 cm^−1^ is observed for p(kA)+p(eA) (Figure 6a). Low intensity FTIR peaks around 1680 cm^−1^ also indicate β-sheet formation [53] and explain the tendency of these complexes to behave more like solids compared to p(kL)+p(eL). The FTIR data shows that p(kG)+p(eG) has more β-sheet content than p(kA)+p(eA). We again attribute this increased tendency for hydrogen bonding for the less hydrophobic residues, to their smaller size being incapable of hindering hydrogen bond formation.

### 2.6. Encapsulation Behavior of Complexes

**Figure 7:**
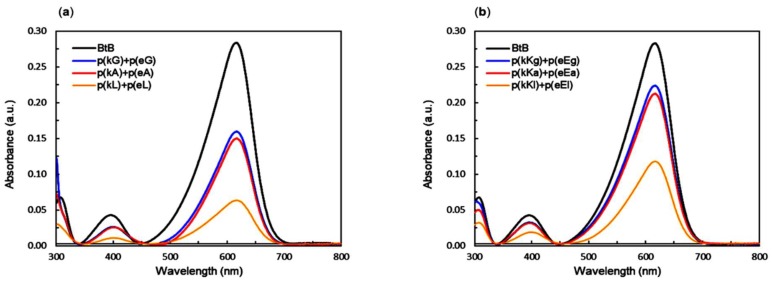
UV-vis curves of BtB in aqueous solution and in the complex supernatant: (**a**) p(kX)+p(eX) complexes; (**b**) p(kKx)+p(eEx) complexes. λ_max_ observed at 617 nm. Total concentration of samples was 5 mM with respect to the monomer charge and 10 µM of the dye. The amount of dye in the supernatant phase decreases with increasing hydrophobicity of the sequence.

In order to study the ability of complexes to encapsulate hydrophobic agents, a nonionic dye containing three aromatic benzene rings, bromothymol blue (BtB), was considered to interact with the PECs. All PEC solutions were prepared at a final concentration of 5 mM with respect to the monomer charge and 10 µM of the dye. Salt was added only to the p(kKx)/p(eEx) complexes in order to increase droplet size for imaging (NaCl: total concentration 40 mM). After complexation of polyelectrolytes with BtB, the samples were centrifuged for 15 min at 10,000 rpm. The supernatant was then carefully removed using a micropipette and transferred into cuvettes (pathlength = 1 cm) for absorbance measurements using a UV-vis spectrophotometer. The absorbance spectra of all supernatants as well as the free dye in solution is shown in Figure 7a,b. They all show a maximum at 617 nm, as expected for BtB at pH 7 [54]. The dye content of each supernatant was calculated using the Beer-Lambert Law, in which the free dye was used to obtain a precise molar extinction coefficient. The amount of dye encapsulated in the different PECs (encapsulation efficiency) is summarized in Table 3 based on the following equation:

(1) $$\mathrm{Encapsulation}\;\mathrm{efficiency}\;\left(\%\right)=100-\frac{\mathrm{amount}\;\mathrm{of}\;\mathrm{dye}\;\mathrm{in}\;\mathrm{the}\;\mathrm{supernatant}\;\mathrm{phase}}{\mathrm{total}\;\mathrm{amount}\;\mathrm{of}\;\mathrm{dye}\;\mathrm{in}\;\mathrm{the}\;\mathrm{system}}$$

**Figure 8:**
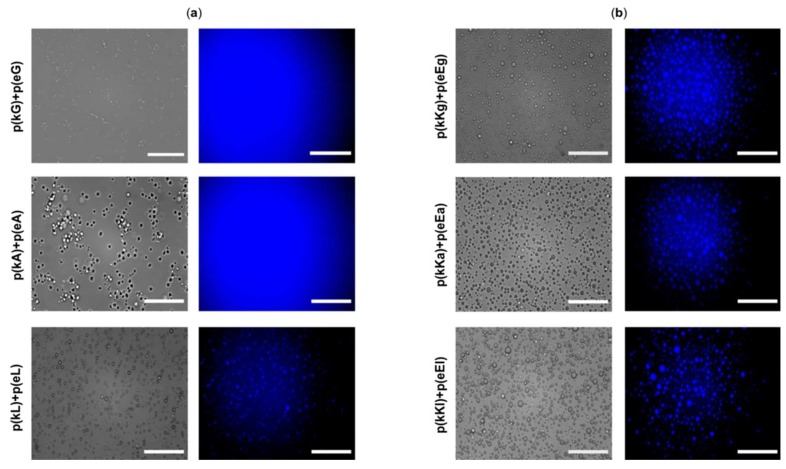
Optical micrographs of complexes (**a**) p(kX)+p(eX)with 15 µM BtB and (**b**) p(kKx)+p(eEx) with 10 µM BtB. Left images are brightfield, while the right images are fluorescence images taken with a DAPI filter that has an excitation wavelength of 377 nm and emission of 447 nm. All images in (**a**) were taken with the same fluorescence intensity in order to visually discern differences in encapsulation. All images in (**b**) were also taken at the same fluorescence intensity, but the intensity of (**a**,**b**) are different. Scale bars, 100 µm.

The absorbance intensity of the supernatants decreases as the hydrophobicity of complexes increases indicating more encapsulation of BtB in the complex phase (Table 4). Hydrophobicity is the driving force for BtB interaction with the PECs, and the enhancement of hydrophobicity within the complex leads to higher sequestration of BtB with more hydrophobic sequences [55]. Overall the p(kX)+p(eX) sequences were able to encapsulate more dye than the p(kKx)+p(eEx) sequences due to their increased hydrophobic content (15 non-charged monomers versus 10 non-charged monomers). Therefore, p(kL)+p(eL) which has the highest hydrophobicity compared to the other complex pairs is expected to have stronger interactions with the hydrophobic dye (BtB) in the complex phase and to show the best encapsulation efficiency as confirmed by the UV-vis measurements (Table 4). Fluorescence imaging of the complexes before centrifugation, as in Figure 8a,b, shows a higher intensity within PECs compared to the background for p(kL)+p(eL) and all 3 p(kKx)+p(eEx) sequences indicating preferential encapsulation to the PEC. This is also a confirmation of the observed UV-vis results indicating the decrease of the dye absorbance in the supernatant phase and the increase of its absorbance in the complex phase by increasing the hydrophobicity. For p(kG)+p(eG) and p(kA)+p(eA), the fluorescence appears uniformly distributed throughout the image, this is likely due to the decreased encapsulation efficiency of these sequences compared to p(kL)+p(eL) and the fact that the same imaging intensity was used for the entire p(kX)+p(eX) series. 

## 3. Materials and Methods 

### 3.1. Materials 

All fluorenylmethyloxycarbonyl (Fmoc)-protected amino acids in this study were purchased from Chem-Impex International Inc. (Wood Dale, IL, USA) and Fmoc-Rink Amide resin (0.32 mmol/g) was purchased from Novabiochem (Burlington, MA, USA). Dimethylfoarmamide (DMF), sodium chloride, urea and trifluoroacetic acid (TFA), were purchased from Fisher Scientific (Fair Lawn, NJ, USA). Piperidine and *N*-methylmorpholine were purchased from Sigma-Aldrich (St. Louis, MO, USA). Bromothymol blue (BtB) was purchased form Alfa Aesar (Ward Hill, MA, USA). 2-(1H-benzotriazol-1-yl)-1,1,3,3-tetramethyluronium hexafluorophosphate (HBTU) was purchased from Oakwood Chemical, (Estill, SC, USA). Triisopropylsilane (TIS) was purchased from Acros (Morris Plains, NJ, USA). 

### 3.2. Peptide Synthesis 

We used a PS3 peptide synthesizer (Gyros Protein Technologies Inc., Tuscan, AZ, USA) to synthesize the hydrophobically patterned peptides using Fmoc-based solid phase peptide synthesis. All patterned peptides contained 30 amino acids and were synthesized on Rink Amide resin. As all amino acids as well as the resin were Fmoc-protected, we used a piperidine solution in DMF (20% *v*/*v*) to remove the Fmoc-protecting group in every step before addition of the next amino acid. 2-(1*H*-benzotriazol-1-yl)-1,1,3,3-tetramethyluronium hexafluorophosphate (HBTU, Oakwood Chemical, Estill, SC, USA) to activate and 0.4 M *N*-methylmorpholine in DMF to deprotonate the carboxylic groups of amino acids were also used in each coupling step. Peptide cleavage from the resin and side chain protecting group removal were performed using a cleavage cocktail of 95% TFA 2.5% TIS and 2.5% water (*v*/*v*)). The peptides were then precipitated in the cold diethyl ether, centrifuged and lyophilized to be ready for further use. 

### 3.3. Preparation of Polypeptide and Complex Solutions 

Stock solutions of 10 mM with respect to the monomer charge were prepared in deionized water for each polypeptide and adjusted to pH 7 using HCl or NaOH solutions as needed. At pH 7 it is assumed that the polypeptides are fully charged (pK_a_ of polyglutamic acid and polylysine is around 4.3 and 10, respectively and at a pH condition of at least two units away from the pK_a_ of a polyelectrolyte, the ionic groups are assumed to be fully charged [2,45,56]). Stock solutions of 2 M sodium chloride and 8 M urea were prepared and adjusted to pH 7.0. All stock solutions were stored at 4 °C and all powder materials were stored at −20 °C. 

Polypeptide complexation was performed with sequential addition of equal amounts of the polyanion (first) and polycation (second) stock solutions, into varying concentrations of salt or urea solutions to a final volume of 400 μL in a microcentrifuge vial (Fisher Scientific, Fair Lawn, NJ, USA). Vortexing was performed after each step of adding a component. Consistency in all sample preparations was considered. 

### 3.4. Characterization and Analysis of Polypeptides and Complex 

#### 3.4.1. Mass Spectroscopy

A Bruker Microflex LRF matrix-assisted laser desorption/ionization time of flight (MALDI-TOF) mass spectrometer (Fremont, CA, USA) was used to identify the actual molecular weight of the peptides on a 96-spot target plate (MSP 96 target ground steel). 

#### 3.4.2. Nuclear Magnetic Resonance (NMR) Spectroscopy

To characterize and confirm the composition and also the structural unit of the polypeptides, H-NMR spectra were recorded on either a Varian VNMRS 500 MHz or Bruker Avance III 400 MHz. To confirm the elimination of TFA from some polycations, fluorine NMR (F-NMR) was carried out, using a Bruker Avance III 400 MHz NMR (Billerica, MA, USA,). 

#### 3.4.3. Secondary Structure Characterization of Polypeptides and Complexes 

A circular Dichroism (CD) spectrophotometer (Olis DMS20, Bogart, GA, USA) was used to confirm the secondary structure and chiral pattern of individual polypeptides as well as the complexes. All measurements were performed at a concentration of 0.2 mg/mL using a cuvette of 1 mm path length in a wavelength range from 190 nm to 250 nm and an average of 5 scans at room temperature. 

A Fourier transform infrared spectrometer (FTIR) spectrometer (Spectrum 100, PerkinElmer, Waltham, MA, USA) with an attenuated total reflectance (ATR) diamond was used for the structural characterization of polypeptide solutions as well as the complexes. All samples were prepared in D_2_O at a final charged monomer concentration of 100 mM. 80 scans from 650 cm^−1^ to 4000 cm^−1^ at a resolution of 4 cm^−1^ were taken at room atmosphere and a background scan was taken and subtracted by the software before each measurement. We subtracted the D_2_O spectrum manually from each data after the measurements. For the p(kX)+p(eX) complexes, which showed solid-like behavior, a higher concentration of 200 mM charged monomer was used and the samples were centrifuged for 15 min at 10,000 rpm. FTIR was performed on the spun down complex phase using only 50 scans.

#### 3.4.4. Turbidity Measurements

A plate reader equipped with an ultraviolet spectrophotometer (Cytation5 imaging reader, Biotek Inc., Winooski, VT, USA) was used for the turbidity measurements. 200 μL of the sample solution was dispensed into a 96 well-plate (Costar, Corning Inc. Kennebunk, ME, USA) right after complexation for the measurement at a wavelength of 500 nm. None of the polypeptides absorb light at this wavelength. The turbidity is defined by T = −ln(I/I_0_), while I_0_ is incident light intensity and I refers to the intensity of the light passed through the sample volume. Turbidity was reported in absorption units (a.u.). Each experiment was repeated three times and the error bars on turbidity plots represent the calculated standard deviation of the data. 

#### 3.4.5. UV-vis Spectroscopy 

A Cary 60 UV-vis spectrophotometer by Aligent Technologies (Santa Clara, CA, USA) was used to collect absorbance spectra of the dye and supernatant solutions at room temperature using a wavelength range of 300 to 800 nm.

#### 3.4.6. Optical Microscopy

Optical microscopy (Cytation5 imaging reader, Biotek Inc., Winooski, VT, USA) of the samples in bright-field mode and 20× magnification was used for obtaining physical images of the complexes in a 96 well-plate. Fluorescence imaging of the complexes with BtB was also carried out with the same instrument using the DAPI filter cube (Biotek Inc., Winooski, VT, USA) that has an excitation wavelength of 377 nm and emission of 447 nm. The BtB concentration for imaging was 15 µM for p(kX)+p(eX) (as we could not get clear images with the dye concentration of 10 µM for this sequence) and 10 µM for p(kKx)+p(eEx). 

## 4. Conclusions

Here, we presented two different designs of hydrophobically patterned ionic polypeptides that were used to study the effect of hydrophobicity on polyelectrolyte complexes. The peptides were designed to have alternating chiral patterns in order to suppress hydrogen bonding interactions and promote complex coacervate formation over solid precipitates. However, the first design containing an alternating pattern of d-charged monomer and l-hydrophobic monomer, p(kX)+p(eX), formed irregularly shaped complexes that transitioned to spherical droplets in the presence of urea, confirming hydrogen bond formation. This solid-like behavior was most prominent for sequences with smaller side chains indicating that larger hydrophobic amino acids incorporated into this pattern could potentially suppress hydrogen bonding completely. This would allow the creation of highly hydrophobic ionic polypeptides that form liquid coacervate phases. The second set of sequences, had an alternating pattern of identically charged d- and l-monomers, which suppressed hydrogen bonding and resulted in liquid coacervate formation, but had a decreased amount of hydrophobic content. 

Overall, the stability of the complexes increased with increasing hydrophobic content, when electrostatic contributions were kept constant. However, peptides designed with increased charge density were much more stable, as expected, due to the increasing electrostatic interactions. By varying the amount of hydrophobic interactions, we showed that we could enhance complex formation with increasing temperature, which normally suppresses complex formation. This mechanism could be used to design new temperature sensitive materials. Moreover, we showed that encapsulation efficiency of a model hydrophobic dye increases with the hydrophobicity of the sequence. This current method of patterning and self-assembly of peptides can provide a promising approach to explore their application as drug delivery carriers that contain both charged biologics, like nucleic acids and proteins, as well hydrophobic drugs. This is especially relevant given that the peptides we designed can be modified to include non-charged polymers that stabilize complex formation on the nanoscale [2,57,58] and can also be decorated with targeting elements [59].

## Figures and Tables

**Table 1:** Side chain of glycine (G), alanine (A) and leucine (L).

Glycine	Alanine	Leucine
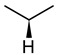	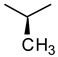	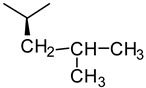

**Table 2:** Chiral patterned peptides. K refers to lysine and E to glutamic acid and X refers to either glycine, alanine or leucine. Lower and upper cases indicate D and L chirality, respectively. Degree of polymerization is 30 for all peptide sequences.

Peptide Patterns	Polycations	Polyanions
First generation	(kX)_15_	(eX)_15_
Second generation	(kKxKkX)_5_	(eExEeX)_5_

**Table 3:** Mass to charge ratio measurements of the patterned peptides using MALDI-TOF.

Peptide	*m*/*z*	Theoretical Mass
(kG)_15_	2810.71	2795.40
(eG)_15_	2825.66	2809.51
(kA)_15_	3021.94	3005.79
(eA)_15_	3064.13	3019.91
(kL)_15_	3665.76	3636.98
(eL)_15_	3698.42	3651.1
(kKg)_10_	3149.27	3150.99
(eEg)_10_	3174.31	3169.82
(kKa)_10_	3296.25	3291.26
(eEa)_10_	3327.89	3310.09
(kKl)_10_	3710.85	3712.05
(eEl)_10_	3752.90	3730.88

**Table 4:** Encapsulation of bromothymol blue (BtB) in polypeptide complexes determined using UV-vis spectroscopy.

Supernatant Solution	Dye Concentration (µM)	Sequestration in the Complex Phase (%)
Bromothymol blue	10	-
p(kG)+p(eG)	5.59	44.1
p(kA)+p(eA)	5.25	47.5
p(kL)+p(eL)	2.15	78.4
p(kKg)+p(eEg)	7.89	21.1
p(kKa)+p(eEa)	7.49	25.1
p(kKl)+p(eEl)	4.12	58.8

## Supplementary Materials

The following are available online, Figure S1: MALDI-TOF mass spectroscopy of the peptide sequences, Figure S2: H-NMR spectroscopy of the peptide sequences, Figure S3: H-NMR spectroscopy of polypeptides after three times lyophilization or dialysis, Figure S4: Circular dichroism (CD) spectroscopy of the peptide sequences, Figure S5: F-NMR spectroscopy of the polycations of the p(kX) sequences.
