# Supplementary material for: Engineering Peptide-Based Polyelectrolyte Complexes with Increased Hydrophobicity
Source: Molecules. 2019 Mar 1;24(5):868. doi: 10.3390/molecules24050868 (PMC6429441; doi:10.3390/molecules24050868)
Supplement: Supplementary file 1 [file molecules-24-00868-s001.pdf]

Supplementary Information

Engineering Peptide-based Polyelectrolyte Complexes with Increased Hydrophobicity

Sara Tabandeh [1] and Lorraine Leon [1,2]*

1 Department of Materials Science and Engineering, University of Central Florida, Orlando, FL, USA;
2 NanoScience Technology Center, University of Central Florida, Orlando, FL, USA;
* Correspondence: lorraine.leon@ucf.edu; Tel.: +1-407-823-5378

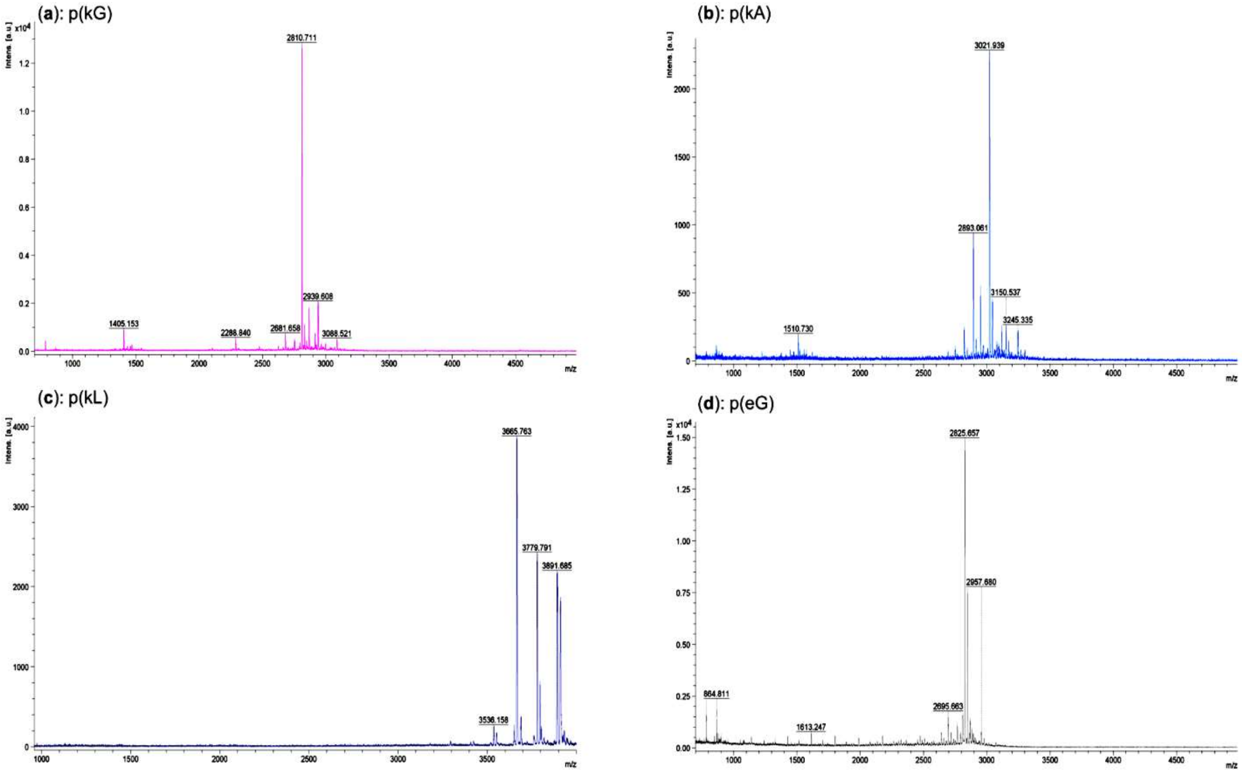

Figure S1. MALDI-TOF mass spectroscopy of the peptide sequences: (a) p(kG); (b) p(kA); (c) p(kL); (d) p(eG).

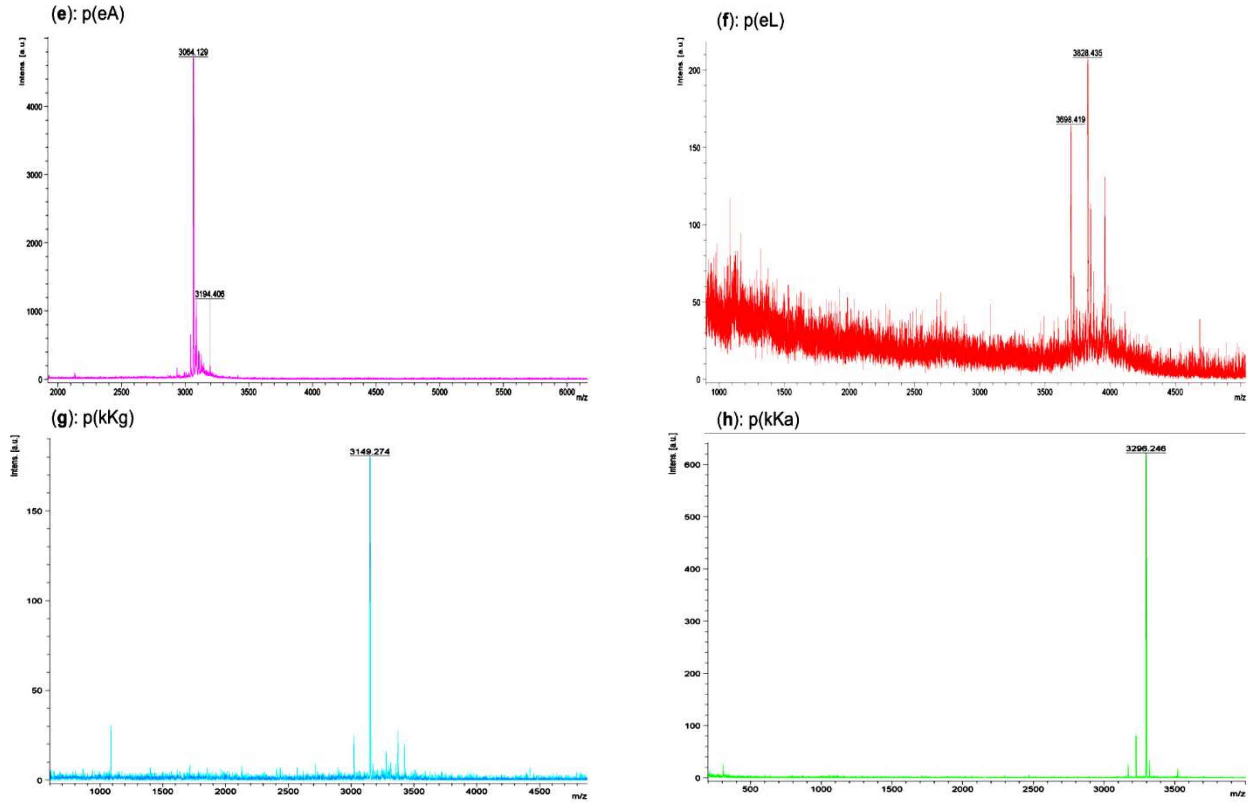

Figure S1. Continued. MALDI-TOF mass spectroscopy of the peptide sequences: (e) p(eA); (f) p(eL); (g) p(kKg); (h) p(kKa).

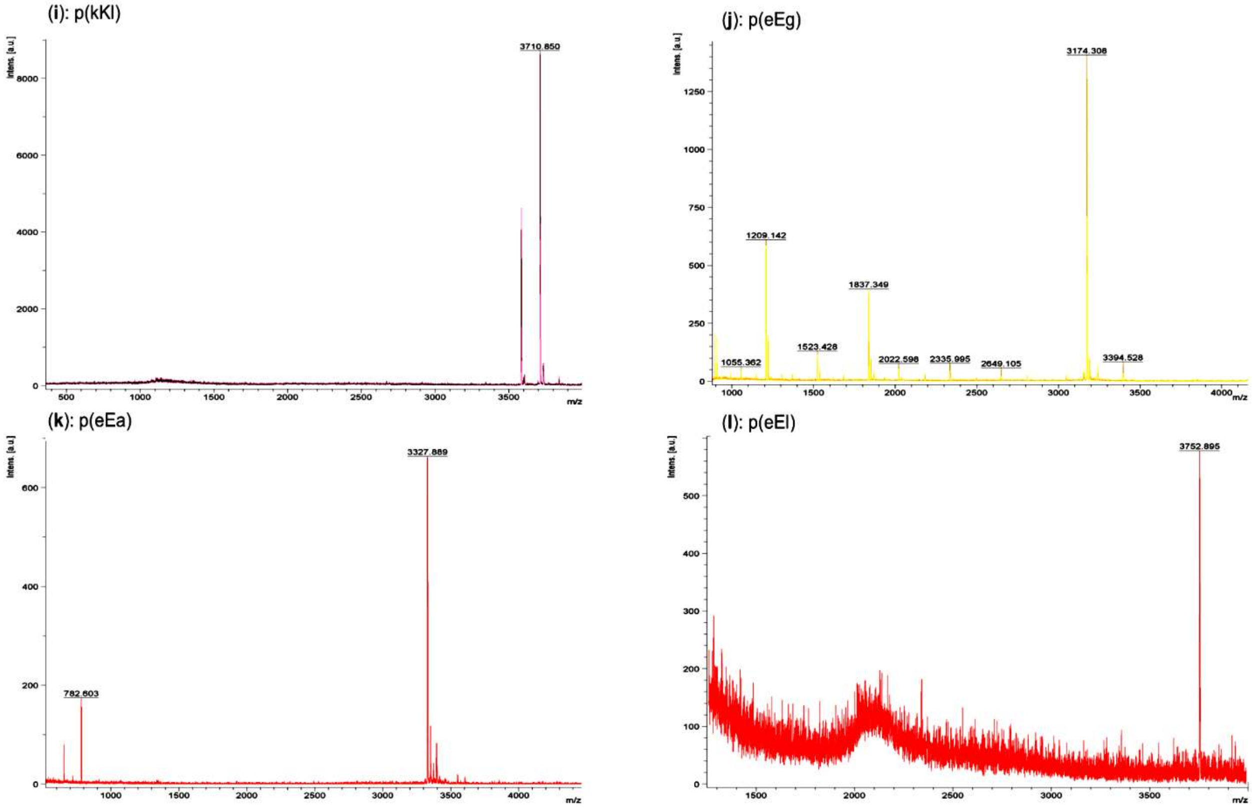

Figure S1. Continued. MALDI-TOF mass spectroscopy of the peptide sequences: (i) p(kKl); (j) p(eEg); (k) p(eEa); (l) p(eEl).

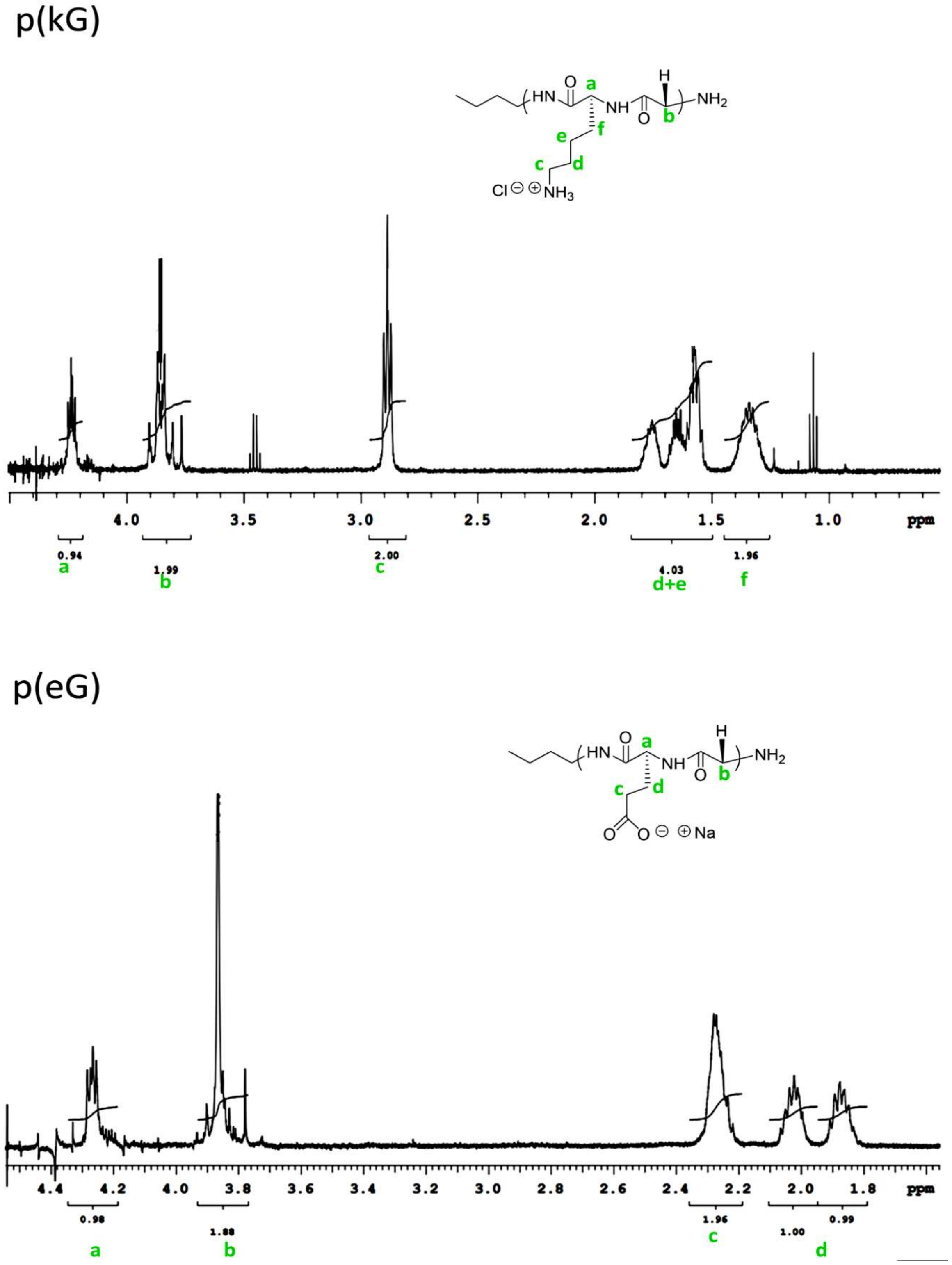

Figure S2. H NMR spectroscopy of the peptide sequences: p(kG) on top; p(eG) on bottom.

p(kA)

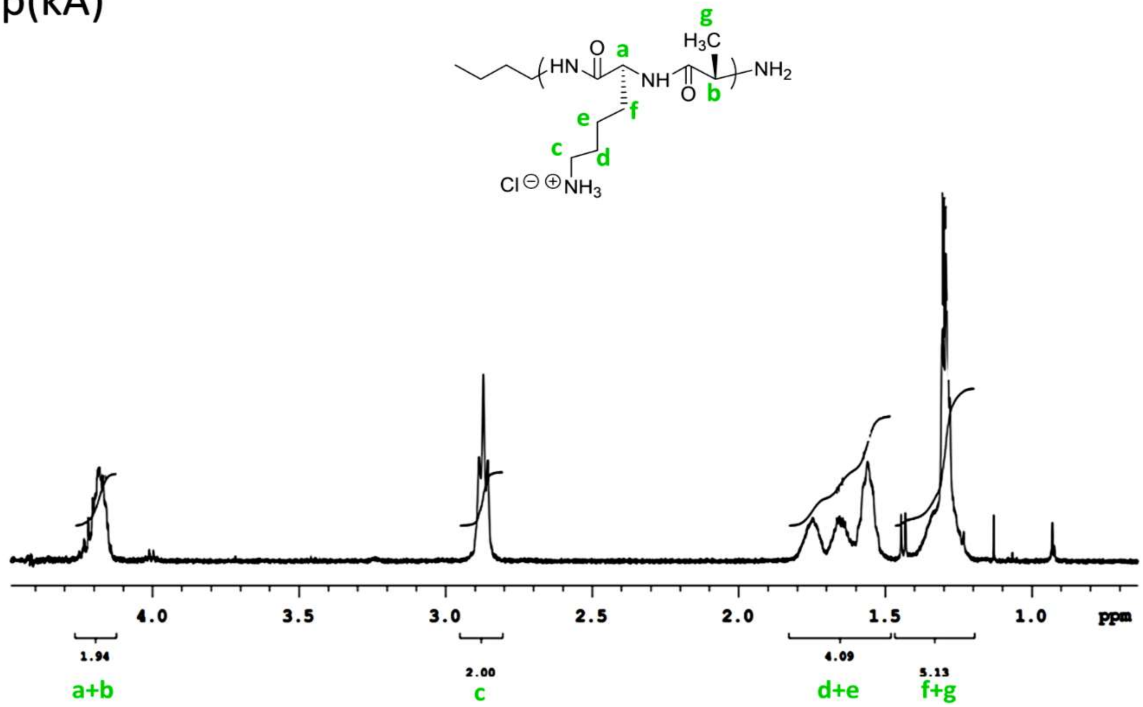

p(eA)

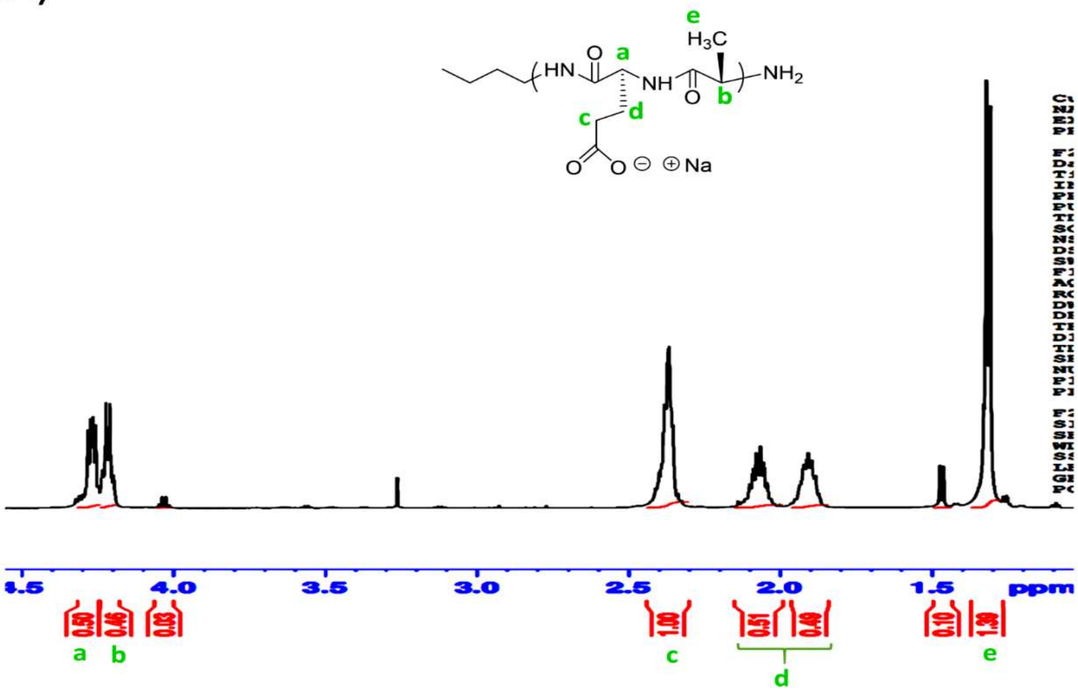

Figure S2. Continued. H NMR spectroscopy of the peptide sequences: p(kA) on top; p(eA) on bottom.

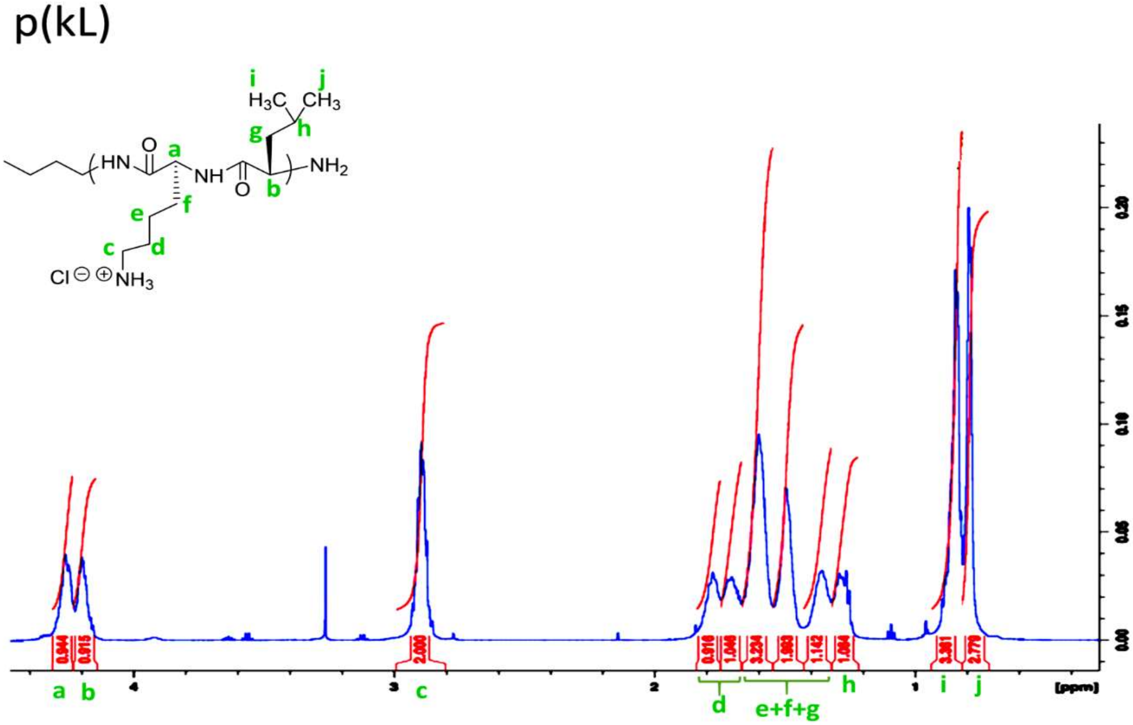

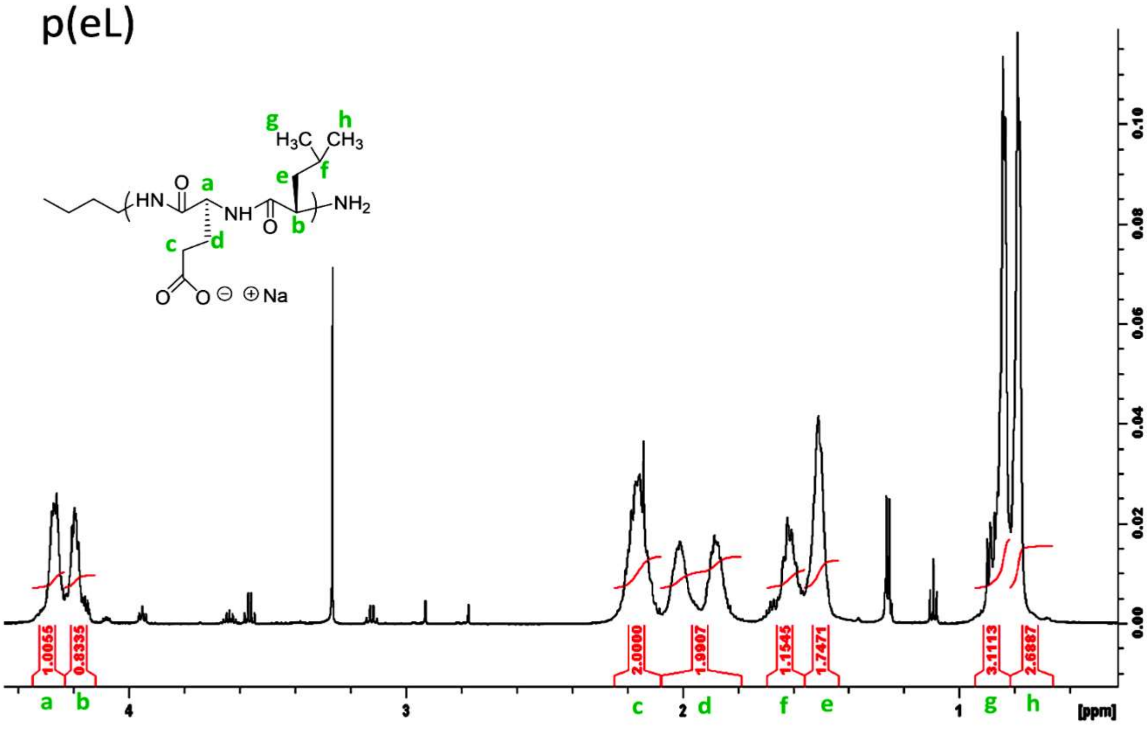

Figure S2. Continued. H NMR spectroscopy of the peptide sequences: p(kL) on top; p(eL) on bottom.

p(kKg)

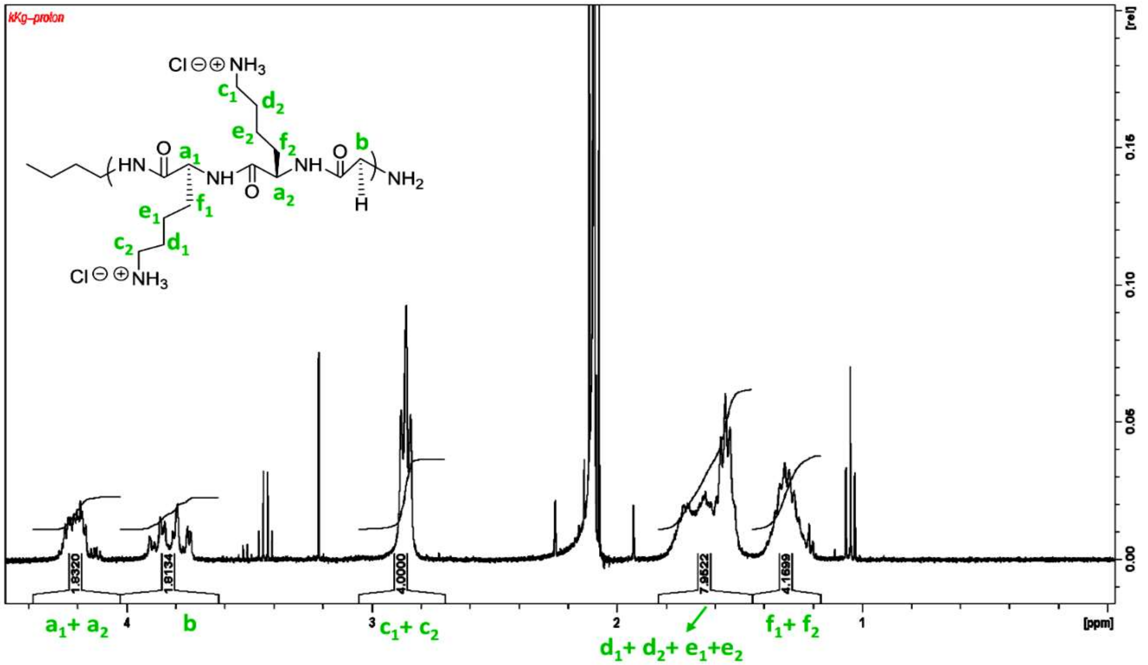

p(eEg)

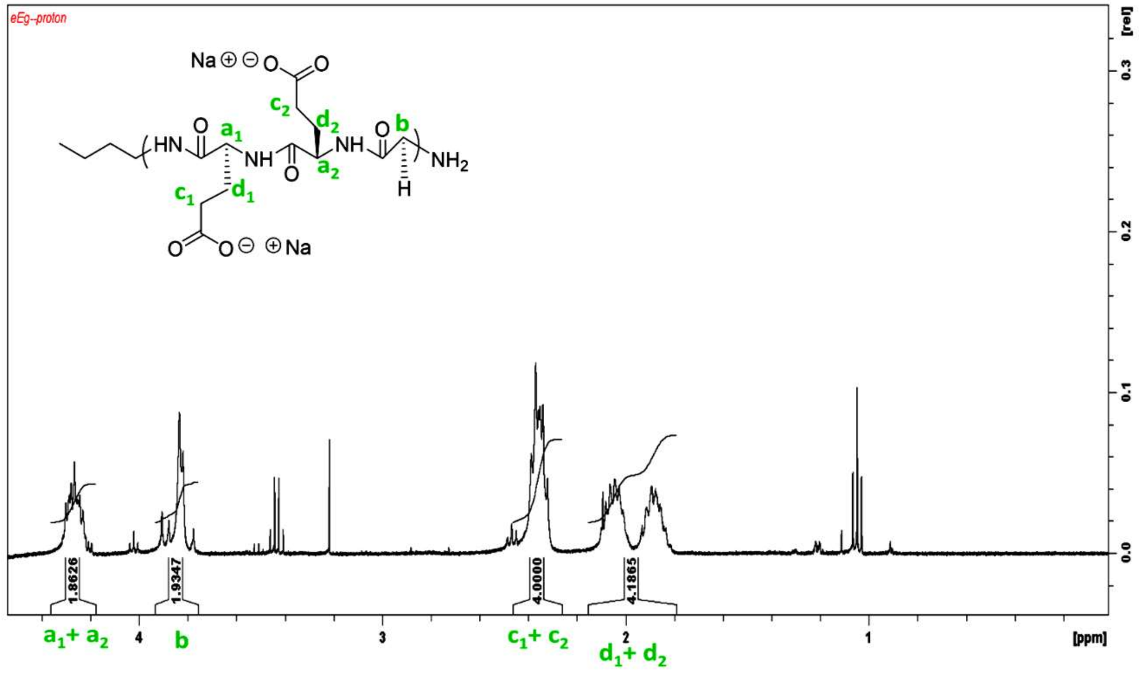

Figure S2. Continued. H NMR spectroscopy of the peptide sequences: p(kKg) on top; p(eEg) on bottom.

p(kKa)

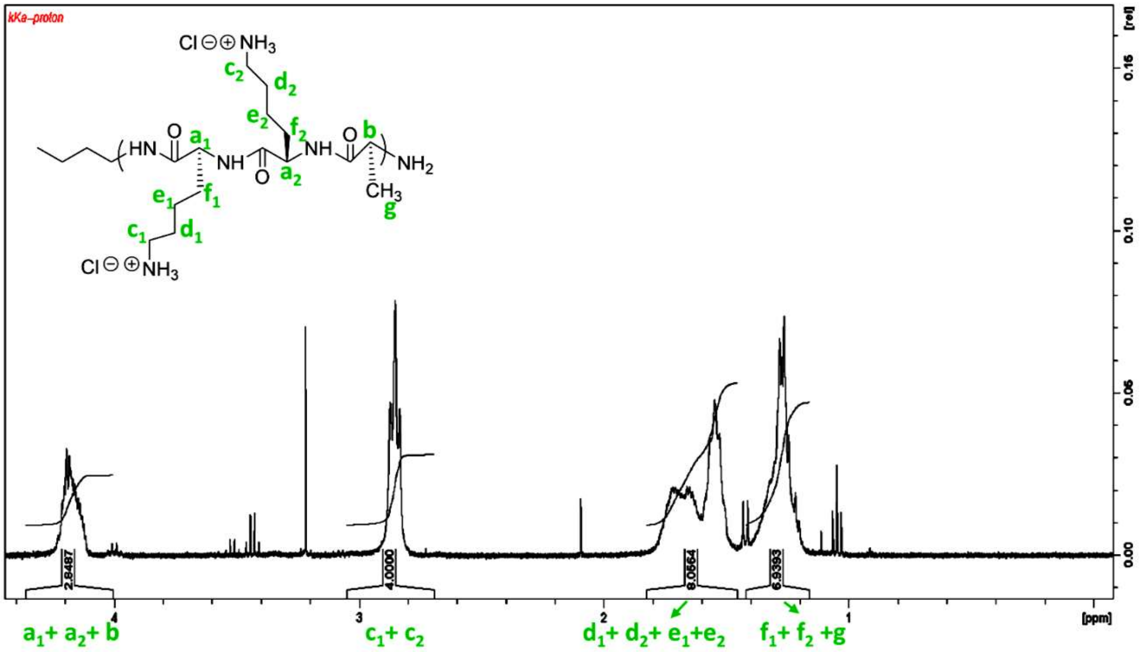

p(eEA)

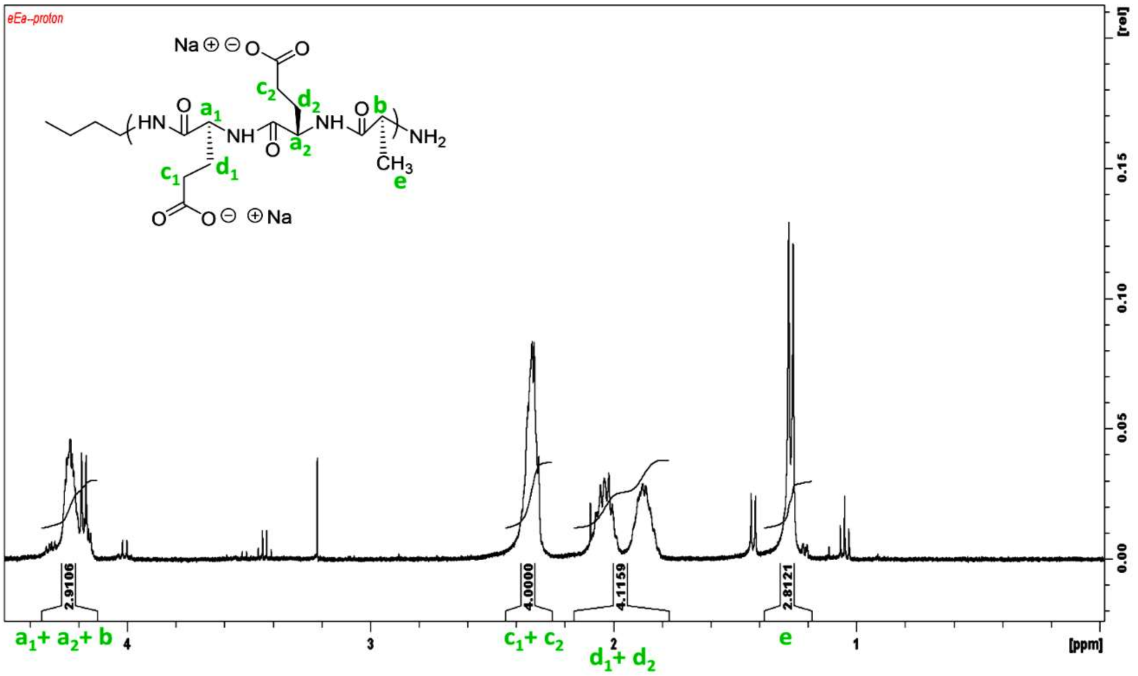

Figure S2. Continued. H NMR spectroscopy of the peptide sequences: p(kKa) on top; p(eEa) on bottom.

p(kKl)

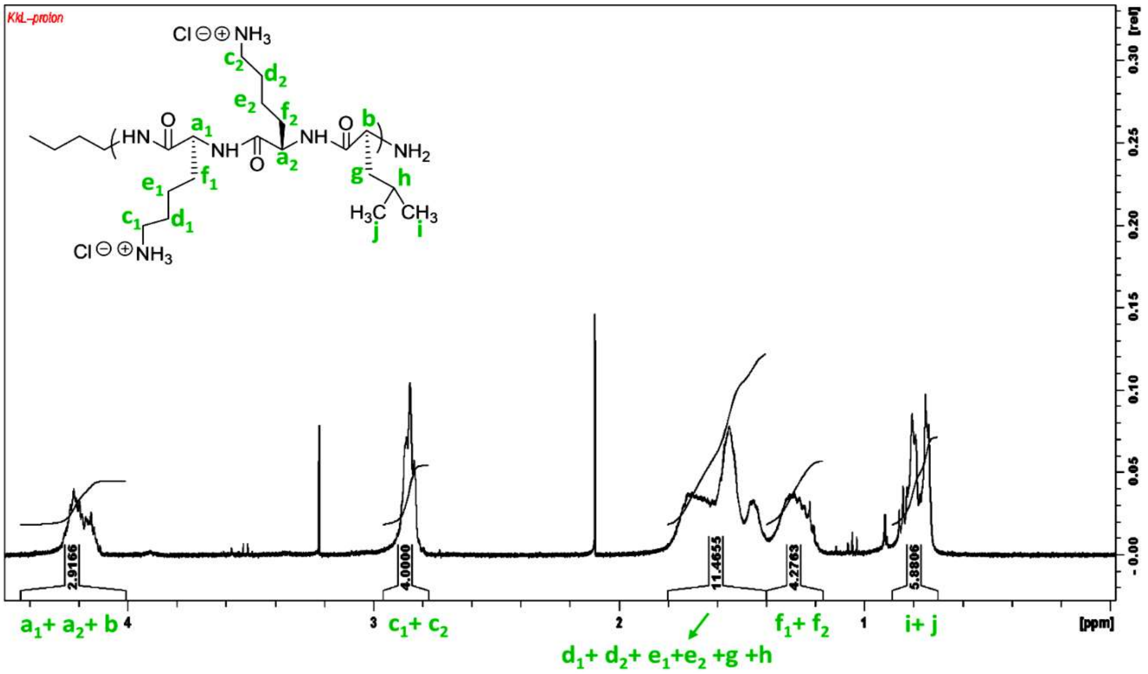

p(eEl)

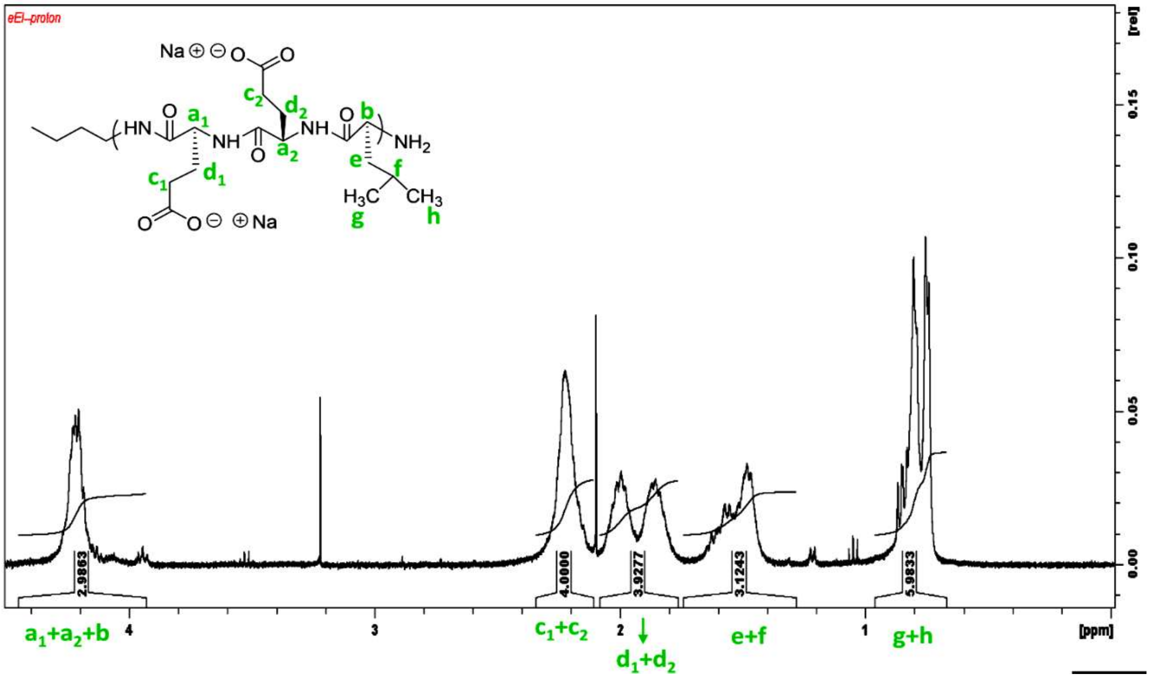

Figure S2. Continued. H NMR spectroscopy of the peptide sequences: p(kKl) on top; p(eEl) on bottom.

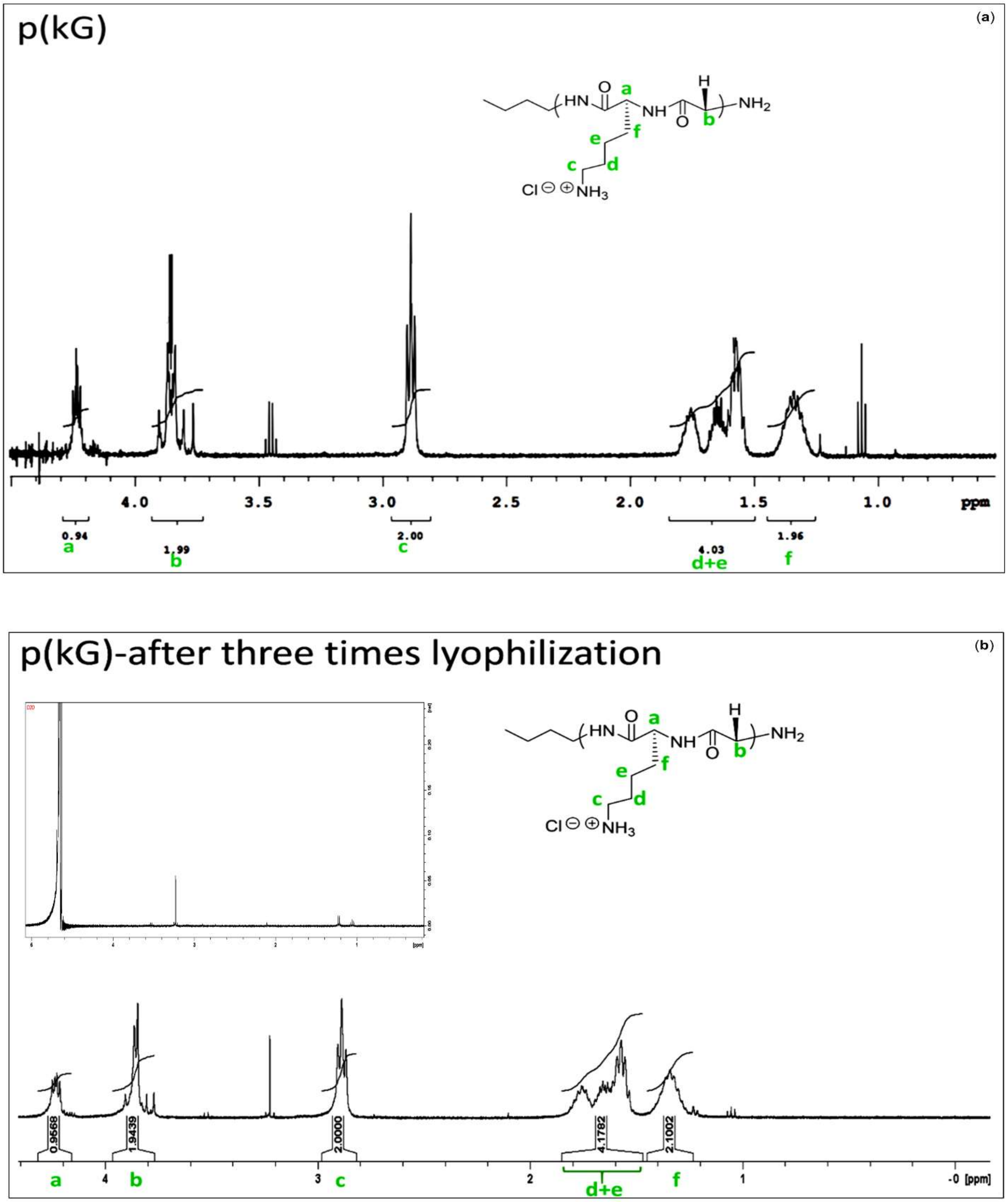

Figure S3. H NMR spectroscopy of polypeptides: (a) p(kG) which shows the signals of diethyl ether at 1.17 and 3.56 ppm for CH_3 and CH_2, respectively; (b) p(kG) after three times lyophilization indicating removal of diethyl ether. Inset: H NMR spectrum of D_2O. The signals around 1 and 3.2 ppm can also be observed in the spectrum of D_2O alone which was used as the solvent for the NMR measurements.

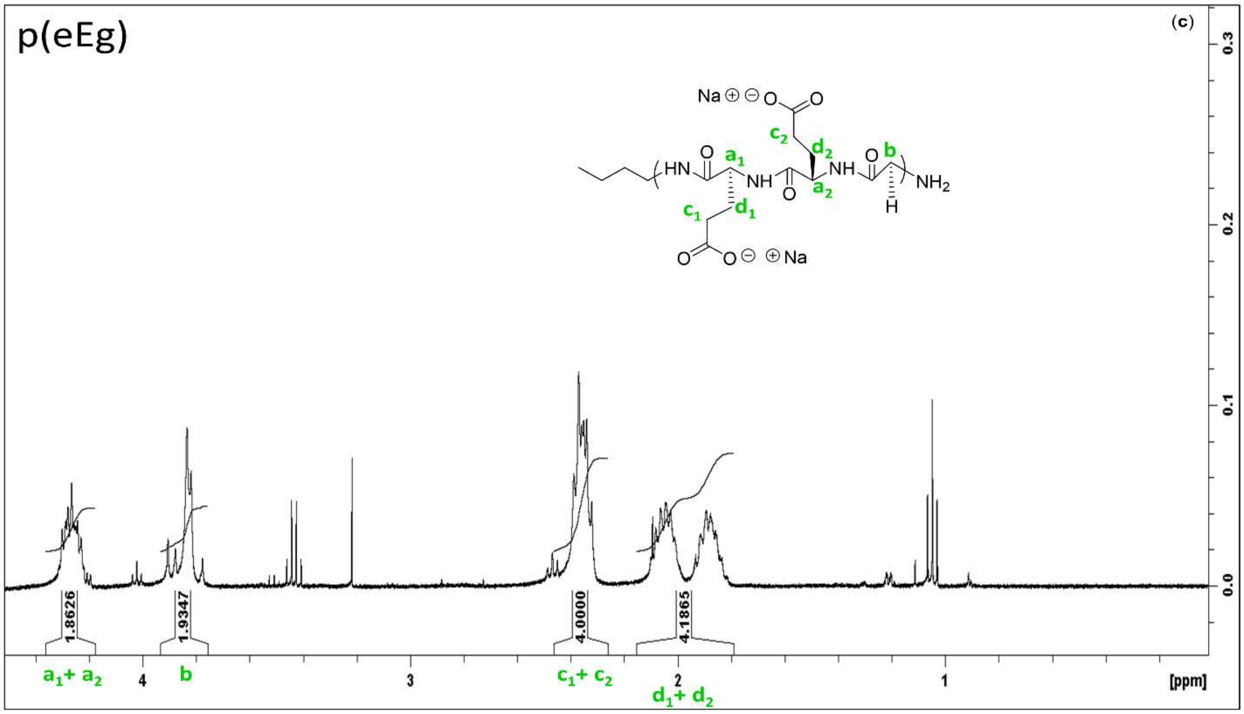

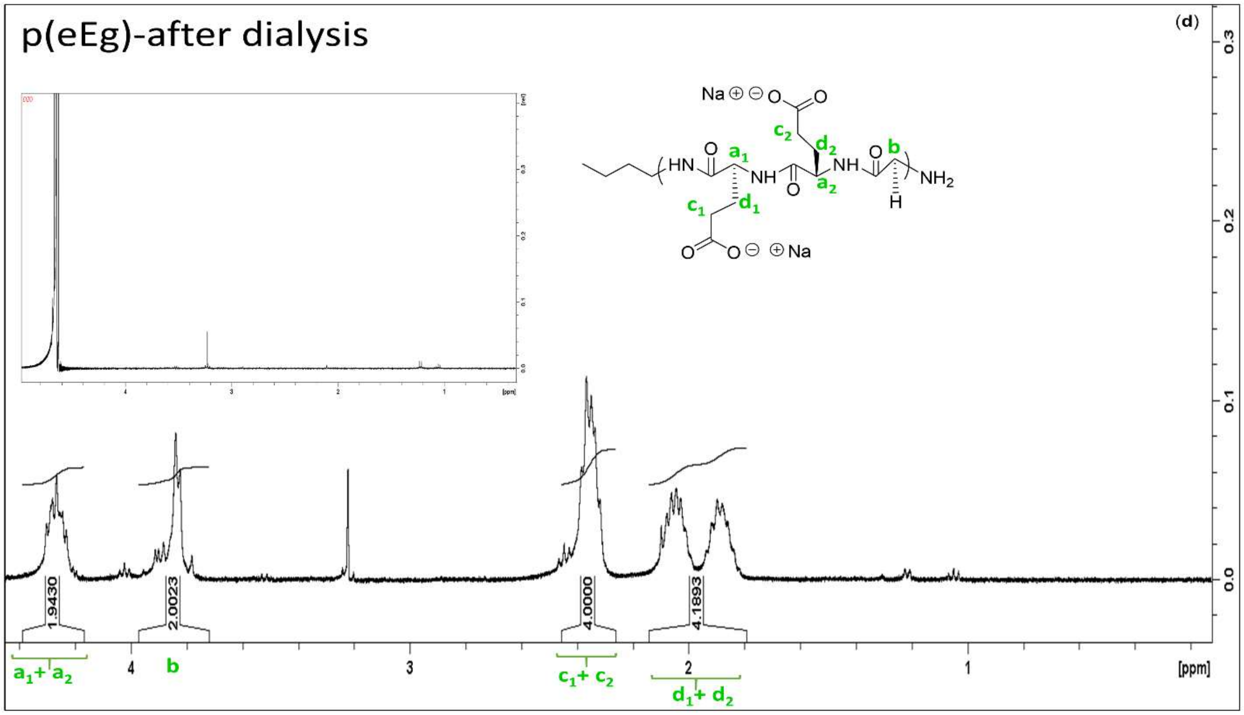

Figure S3. Continued. H NMR spectroscopy of polypeptides: (c) p(eEg) which shows the signals of diethyl ether at 1.17 and 3.56 ppm for CH_3 and CH_2, respectively; (d) p(eEg) after dialysis indicating removal of diethyl ether. Inset: H NMR spectrum of D_2O. The signals around 1 and 3.2 ppm can also be observed in the spectrum of D_2O alone which was used as the solvent for the NMR measurements.

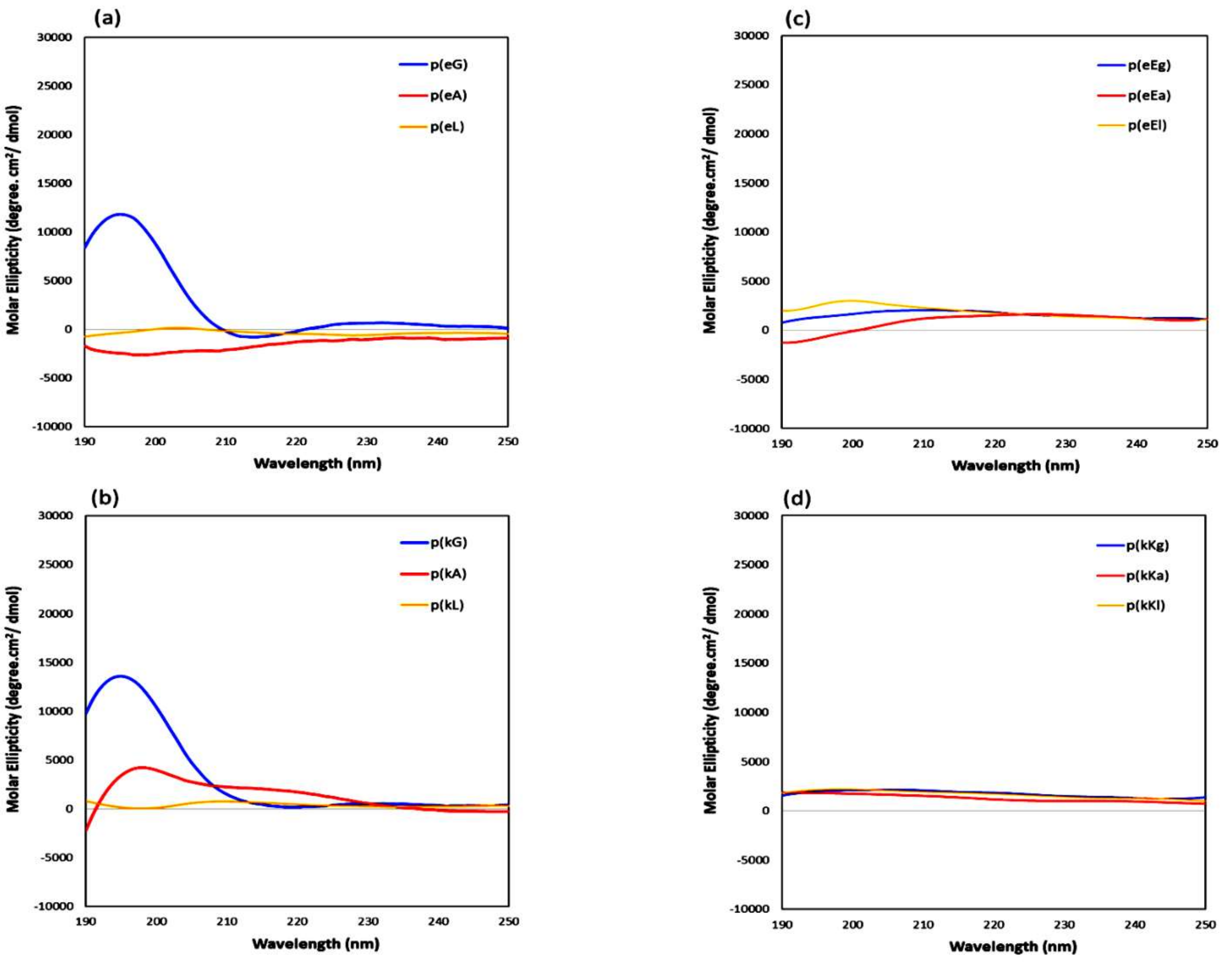

Figure S4. Circular dichroism (CD) spectroscopy of the peptide sequences: (a) p(eX); (b) p(kX); (c) p(eEx); (d) p(kKx).

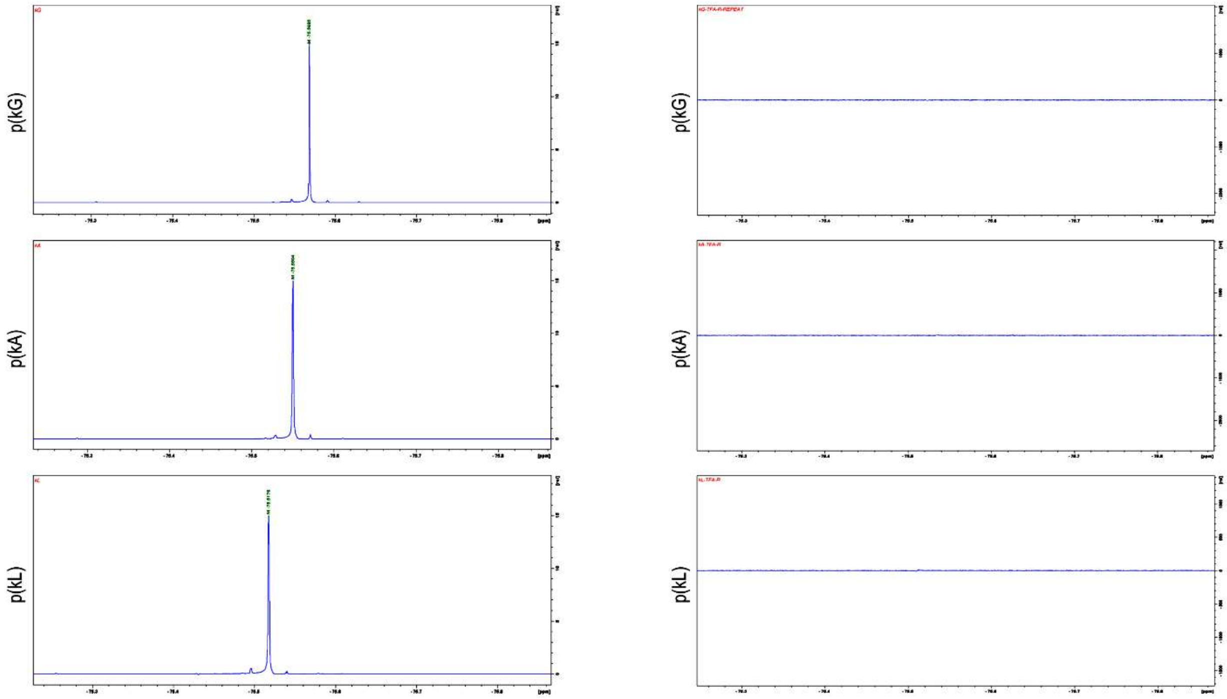

Figure S5. F NMR spectroscopy of the polycations of p(kX) sequence: left side of the image shows the spectra before TFA-elimination; right side of the image shows the spectra after TFA-elimination.
